# Electrochemical Assessment of Mitigation of *Desulfovibrio ferrophilus* IS5 Corrosion against N80 Carbon Steel and 26Cr3Mo Steel Using a Green Biocide Enhanced by a Nature-Mimicking Biofilm-Dispersing Peptide

**DOI:** 10.3390/antibiotics12071194

**Published:** 2023-07-15

**Authors:** Lingjun Xu, Pruch Kijkla, Sith Kumseranee, Suchada Punpruk, Tingyue Gu

**Affiliations:** 1 Department of Chemical & Biomolecular Engineering, Institute for Corrosion and Multiphase Technology, Ohio University, Athens, OH 45701, USA; 2 PTT Exploration and Production, Bangkok 10900, Thailand

**Keywords:** microbiologically influenced corrosion, carbon steel, chromium steel, sulfate-reducing bacteria, biocide enhancer, antimicrobial

## Abstract

MIC (microbiologically influenced corrosion) is problematic in many industries, especially in the oil and gas industry. In this work, N80 carbon steel for pipelines was tested with 26Cr3Mo chromium pipeline steel for comparison in SRB (sulfate-reducing bacterium) MIC mitigation using a THPS (tetrakis hydroxymethyl phosphonium sulfate)-based commercial biocide (Biotreat 5475 with 75–80% THPS by mass). Peptide A, a nature-mimicking synthetic cyclic peptide (cys-ser-val-pro-tyr-asp-tyr-asn-trp-tyr-ser-asn-trp-cys) with biofilm dispersal ability was used as a biocide enhancer. Metal coupons covered with 3-d old *Desulfovibrio ferrophilus* IS5 biofilms were immersed in different biocide solutions. After 1-h treatment, 200 ppm Biotreat 5475, 200 ppm Biotreat 5475 + 200 nM (360 ppb) Peptide A, and 400 ppm Biotreat 5475 achieved 0.5-log, 1.7-log and 1.9-log reductions in sessile cell count on N80, and 0.7-log, 1.7-log, and 1.8-log on 26Cr3Mo, respectively. The addition of 200 nM Peptide A cut the THPS biocide dosage by nearly half. Biocide injection tests in electrochemical glass cells after 1 h exhibited 15%, 70%, and 72% corrosion inhibition efficiency (based on corrosion current density) on N80, and 27%, 79%, 75% on 26Cr3Mo, respectively. Linear polarization resistance and electrochemical impedance spectrometry results also indicated antimicrobial efficacies.

## 1. Introduction

N80 carbon steel is a common pipeline steel in oilfield applications. Like other carbon steels, N80 is susceptible to different types of corrosion such as CO_2_ (carbon dioxide) corrosion and microbiologically influenced corrosion (MIC) [1,2,3,4]. Low chromium steels have much higher corrosion resistance to CO_2_ and they are more economical than stainless steels [5,6,7]. The corrosion product films on chromium steels serve as a passive layer to hinder the outward diffusion of Fe^2+^, and the enrichment of Cr prevents corrosive anions from attacking the steel surface [8,9,10]. The addition of Cr in steels also improves MIC through the passive film a certain degree of antimicrobial effect of Cr ions [11]. 26Cr3Mo is a new grade chromium steel that belongs to 3Cr-L80 series pipeline steels [12].

MIC was first identified a century ago [13]. It is a major issue in the oil and gas industry due to its contribution to equipment failures including pipelines, and pressure vessels [14,15,16]. MIC is reported to be responsible for more than 20% of the total corrosion costs [17,18]. Sulfate-reducing bacteria (SRB) are involved in the majority of severe MIC cases. SRBs are ubiquitous in oilfield operations, because sulfate is widely present in many oilfield operations owing to the use of seawater injection in enhanced oil recovery [19,20,21,22]. To mitigate SRB MIC, biocide treatment is a commonly used technique in oilfields [23,24] together with pigging.

SRB MIC of Fe^0^ (elemental iron) is known to belong to extracellular electron transfer-MIC (EET-MIC) in which sessile cells utilize electrons from extracellular Fe^0^ oxidation for intracellular sulfate reduction in energy production [25]. The following half-reactions can be used to analyze the bioenergetics of the SRB corrosion process with sulfate serving as the terminal electron acceptor [25].

4Fe → 4Fe^2+^ + 8e^−^ (E° = −447 mV_SHE_) (1)

SO_4_^2−^ + 9H^+^ + 8e^−^ → HS^−^ + 4H_2_O (E°’ = −217 mV_SHE_) (2)

The combined reaction has a positive cell potential of +230 mV (at 1 M solutes except H^+^, and pH 7 as indicated by the apostrophe in E). This means this thermodynamically favored corrosion process generates energy that can benefit SRB metabolism. More sessile cells harvest more electrons from Fe^0^. Numerous studies demonstrated that in SRB MIC of Fe^0^, there was a direct correlation showing more sessile cells corresponding to higher weight loss [1]. Thus, in EET-MIC, treating biofilms to reduce the sessile cell counts is the key to MIC mitigation.

When metabolite-MIC (M-MIC) is involved, a biofilm such as an acid-producing bacteria (APB) biofilm can produce local high acidity because volumetric sessile cell density is much higher than planktonic cell density. Thus, mitigating M-MIC also requires biofilm treatment.

Biofilms provide embedded sessile cells with protection from antimicrobial agents [26,27,28]. A much higher dosage of biocide is needed to treat sessile cells compared with planktonic cells [29,30,31]. As a result, traditional biocide dosing suffers from high operational costs and potential environmental issues [32,33]. A mixed-culture field biofilm can become more resistant to a certain biocide after using it for a period of time, because the killing of vulnerable microbes leaves a nutritional niche for more resistant microbes to move in from the surrounding environment [34,35,36]. This leads to a decrease of biocide efficacy and a demand for higher dosages to mitigate the biofilms [37,38].

Environmental-friendly biocides with minimal or no toxicity are preferred in mitigating MIC. Tetrakis hydroxymethyl phosphonium sulfate (THPS) is a popular green biocide in oilfield operations [39,40]. THPS degrades to trihydroxymethyl phosphine [41], which can effectively reduce disulfide bonds in disulfide amino acids in microbial cell walls, leading to cleavage of the bonds and destruction of the cell walls [42,43]. The selective action of THPS can also affect the hNRB (heterotrophic nitrate-reducing bacteria) and so-NRB (sulfide-oxidizing-NRB) activities, resulting in SRB growth inhibition and preventing sulfide formation [44]. Other than killing or inhibiting SRB, THPS can scavenge H_2_S (hydrogen sulfide) in a chemical reaction to mitigate souring [45].

In field operations, green biocide enhancers can be added to biocides to improve biocide efficacy as well as to minimize environmental impact. A small amount of biocide enhancer can significantly improve biocide efficacy or lower the biocide dosage [37]. It was reported that 50 ppm D-amino acid mixtures enhanced 15 ppm THPS to achieve a similar efficacy as 30 ppm THPS against an oilfield biofilm consortium in a lab test [46]. Peptide A is a chemically synthesized 14-mer circular peptide (cys-ser-val-pro-tyr-asp-tyr-asn-trp-tyr-ser-asn-trp-cys). Its core 12-mer sequence was inspired by a biofilm-dispersing protein secreted by a sea anemone to maintain its biofilm-free exterior [47]. Peptide A was reported to be an effective non-biocidal green biocide enhancer against an oilfield biofilm consortium [48]. It was found that in MIC of the oilfield biofilm consortium on C1018 carbon steel, the combination of 100 ppm DBNPA (2,2-dibromo-3-nitrilopropionamide) + 100 nM Peptide A achieved 0.9-log, 0.8-log and 0.6-log further reductions in sessile cell count for SRB, APB (acid-producing bacteria) and GHB (general heterotrophic bacteria), respectively, compared to treatment with 100 ppm DBNPA alone [47]. It was reported that Peptide A alone was not biocidal at or below 100 nM dosage, but when it is used together with a biocide, it exhibited a biofilm dispersal effect which enhanced the biocide efficacy [48].

Chromium steels provide good passivation against CO_2_ corrosion. However, their passive films are (semi-)conductive, allowing an SRB biofilm to harvest electrons across the passive film. Once there is a defective or damaged passive film spot, the spot will be preferentially used for Fe^2+^ outward diffusion in a large cathode-small anode scenario, leading to classical pitting or pit amplification [49]. It was found that after a 7-d incubation with *Desulfovibrio ferrophilus*, 13%Cr had a weight loss of 4.4 mg/cm^2^ (0.30 mm/a uniform corrosion rate) while its maximum pit depth reached 288 μm (15 mm/a pitting rate), compared to a much larger weight loss of 15.2 mg/cm^2^ (1.0 mm/a uniform corrosion rate) accompanied by a much lower maximum pit depth of 7.3 μm (0.38 mm/a pitting rate) for N80 which had much inferior FeS passivation to the passivation provided by Cr oxides/hydroxides on 13%Cr [49]. This means that to deploy a Cr steel for CO_2_ corrosion resistance applications, an MIC assessment and mitigation plan should be in place because there is potential for severe MIC attack.

Before a biocide is field tested, lab testing is desired to probe dosage and efficacy. There are two kinds of biocide tests. One is the biofilm prevention test, in which treatment chemicals are added to the culture medium upon inoculation [48]. The other is the biofilm kill (or biofilm eradication) test that uses biocides to treat pre-established biofilms [46,50,51]. In pipelines, the kill test is relevant, in which a biocide liquid “plug” moves downstream between two pigs driven by pressure. The residence time is rather short. In lab tests, such a short-term (e.g., 0.5 h or 1 h) will not generate a measurable weight loss or pit depth difference, unlike in biofilm prevention tests that last for days. Thus, real-time or near-real-time corrosion rate measurements are needed to calculate corrosion rate changes for the calculation of biocide corrosion inhibition efficiency that can be used to support biofilm sessile cell count reduction. Only electrochemical tests can provide near-real-time transient corrosion rate measurements. OCP (open circuit potential), LPR (linear polarization resistance), EIS (electrochemical impedance spectroscopy), and PDP (potentiodynamic polarization) scans are commonly used electrochemical techniques to assess corrosion [52,53,54].

This work investigated the mitigation efficacy of a THPS-based biocide, namely Biotreat 5475, enhanced by Peptide A against SRB MIC on N80 and 26Cr3Mo. The sessile cell reductions after a 1-h biocide treatment, and electrochemical responses to biocide injection were examined. Because sessile cells in a mature biofilm are more difficult to treat than preventing a biofilm from establishing on a metal surface, relatively high dosages (200 ppm and 400 ppm) of Biotreat 5475 were employed.

## 2. Results and Discussion

### 2.1. Enumeration of Sessile Cells on Coupons Soaked in Petri Dishes with and without Biocide

**Figure 1:**
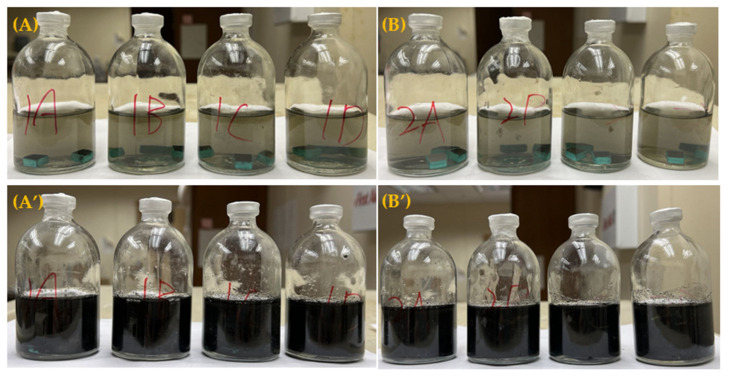
Anaerobic vials containing N80 coupons (**A**,**A’**) and 26Cr3Mo (**B**,**B’**) before and after 3-d incubation with *D. ferrophilus* in EASW (no biocide) at 28 °C.

**Figure 2:**
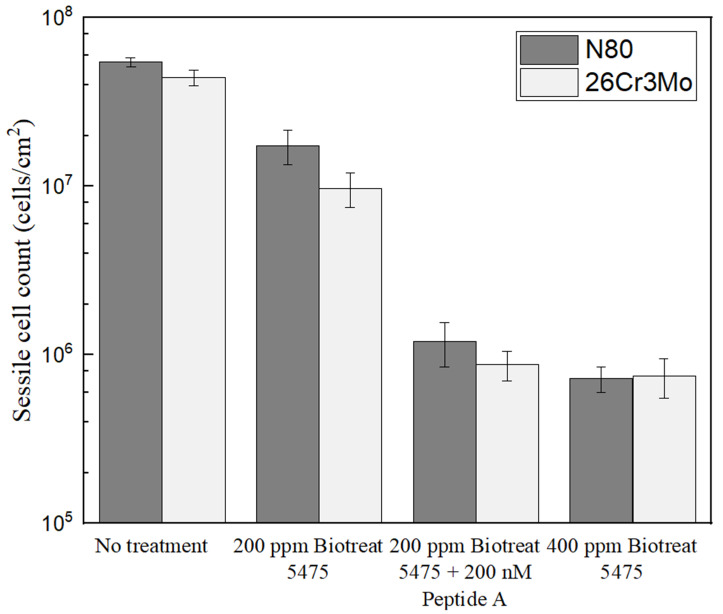
Sessile cell counts on N80 and 26Cr3Mo coupon 1 h after biocide injection into 3-d SRB broths. (Each data point is the average from two replicate coupons).

The anaerobic vials containing N80 and 26Cr3Mo coupons before and after the 3-d incubation are shown in Figure 1. All the vials turned black due to FeS precipitation, indicating healthy SRB growth. Figure 2 demonstrates sessile cell count results on N80 and 26Cr3Mo after the 1-h biofilm kill test (soaking test). The initial sessile cell count on N80 after the 3-d incubation was (5.5 ± 0.3) × 10^7^ cells/cm^2^. After the 1-h treatment with 200 ppm Biotreat 5475, 200 ppm Biotreat 5475 + 200 nM Peptide A, and 400 ppm Biotreat 5475, the sessile cell counts were reduced to (1.8 ± 0.4) × 10^7^ cells/cm^2^, (1.2 ± 0.4) × 10^6^ cells/cm^2^, and (7.3 ± 1.3) × 10^5^ cells/cm^2^, respectively. Thus, 200 ppm Biotreat 5475 only achieved a 0.5-log reduction in sessile cells, while 200 ppm Biotreat 5475 + 200 nM Peptide A, and 400 ppm Biotreat 5475 achieved 1.7-log and 1.9-log sessile reduction. The difference between 200 ppm Biotreat 5475 + 200 nM Peptide A and 400 ppm Biotreat 5475 was not statistically significant (*p* value = 0.25 >> 0.05). Thus, 200 nM Peptide A achieved an enhancement effect of extra 1.2-log sessile cell reduction. It effectively lowered the THPS dosage from 400 ppm to 200 ppm.

For 26Cr3Mo, the initial sessile cell count found after the 3-d incubation was (4.4 ± 0.5) × 10^7^ cells/cm^2^. After the 1-h treatment with 200 ppm Biotreat 5475, 200 ppm Biotreat 5475 + 200 nM Peptide A, and 400 ppm Biotreat 5475, sessile cell counts were reduced to (9.8 ± 2.2) × 10^6^ cells/cm^2^, (8.8 ± 1.8) × 10^5^ cells/cm^2^, and (7.5 ± 2.0) × 10^5^ cells/cm^2^, respectively. These reductions in sessile cells were equivalent to 0.7-log, 1.7-log, and 1.8-log, respectively. For 200 ppm Biotreat 5475 + 200 nM Peptide A and 400 ppm Biotreat 5475, there was no significant statistical difference (*p* value = 0.57 >> 0.05). Thus, 200 nM Peptide A achieved an enhancement effect of extra 1.0-log sessile cell reduction, and it cut THPS dosage by nearly half. Sessile cells on 26Cr3Mo were slightly fewer than those on N80 under the same conditions, which can be explained by the inhibition of microbial growth due to some alloying metal elements such as Cr, Mo in 26Cr3Mo [55].

### 2.2. Electrochemical Test Results

**Figure 3:**
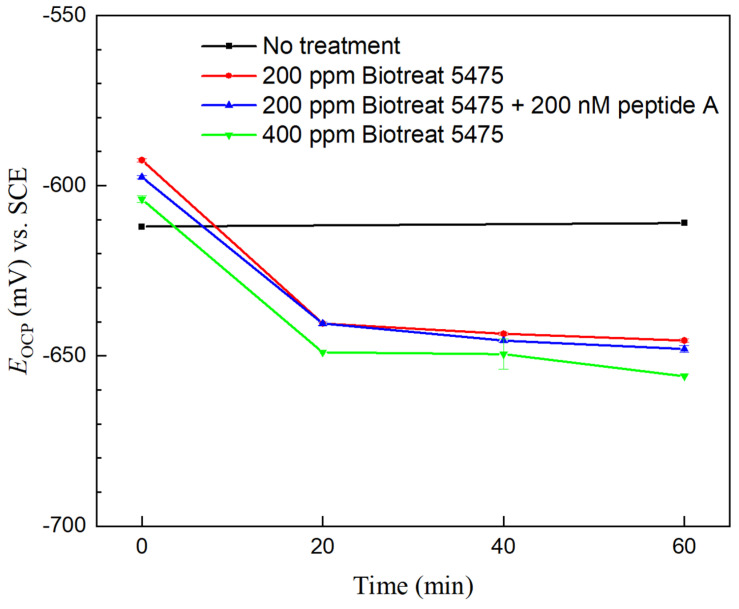
N80 OCP vs. time during 1 h time after biocide injection into 3-d SRB broth. (Range bars were from two replicate scans using two coupons in the same working electrode Epoxy cake).

**Figure 4:**
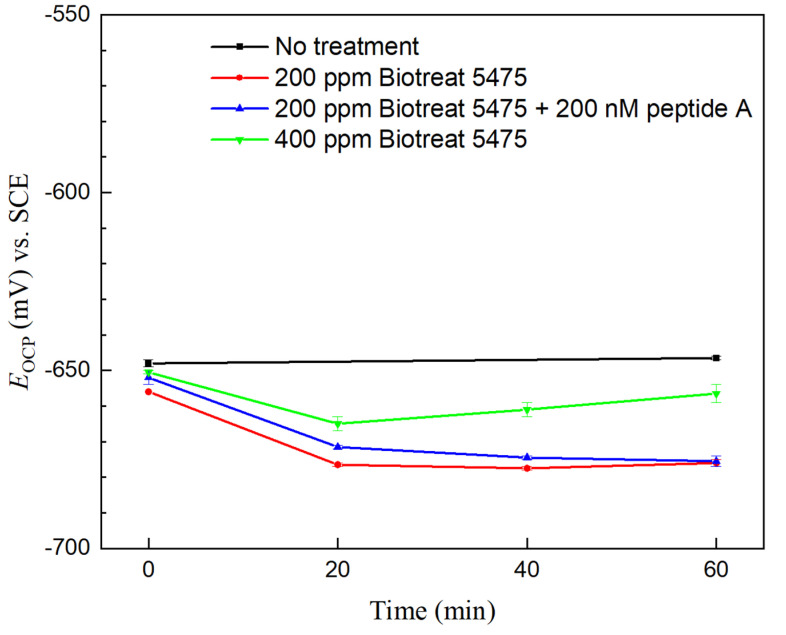
26Cr3Mo OCP vs. time during 1 h time after biocide injection into 3-d SRB broth. (Range bars were from two replicate scans using two coupons in the same working electrode Epoxy cake).

OCP responses of N80 and 26Cr3Mo during the 1 h after the biocide injection treatment are shown in Figure 3 and Figure 4. Without biocide injection, there were very few variations in OCP for N80 and 26Cr3Mo (controls). After biocide injection, OCP values of both N80 and 26Cr3Mo exhibited a decreasing trend over the 1-h period. This is obviously misleading. Theoretically, a lower OCP value indicates a greater corrosion tendency (working electrode losing electrons). However, in most SRB MIC cases, OCP trends are wrong because OCP only indicates corrosion tendency, not the actual corrosion kinetic process or corrosion outcome [47]. Kinetic electrochemical measurements which have been repeatedly proven reliable should be depended upon instead [25,48,52].

**Figure 5:**
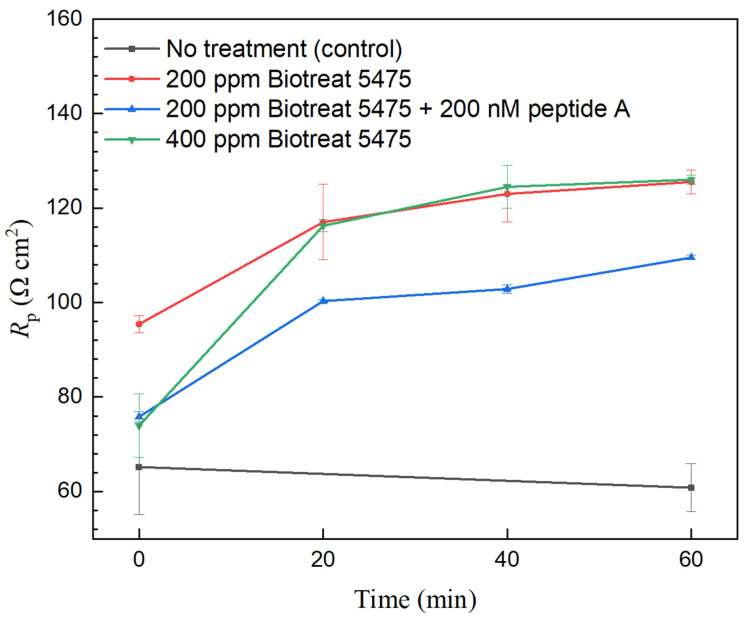
Variations of N80 *R*_p_ from LPR during 1-h time after biocide injection. (Range bars were from two replicate scans using two coupons in the same working electrode Epoxy cake).

**Figure 6:**
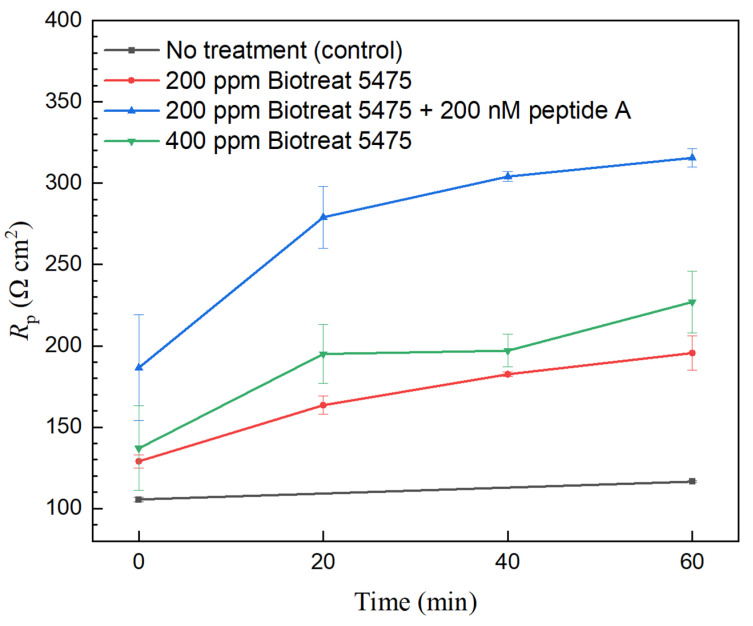
Variations of 26Cr3Mo *R*_p_ from LPR during 1-h time after biocide injection. (Range bars were from two replicate scans using two coupons in the same working electrode Epoxy cake).

Figure 5 and Figure 6 show the polarization resistance (*R*_p_) variation during the 1-h biocide treatment. Because the inverse of *R*_p_ from LPR can be used to represent corrosion rate, the decrease of 1/*R*_p_ can estimate corrosion inhibition efficacy for a biocide treatment,

(3) $$\eta_{i,\mathrm{LPR}}=\left(1-\frac{R_{p,o}}{R_{p}}\right)\times 100\%$$

In the no-biocide injection control glass cell, there were only small variations in *R*_p_. After biocide injection, *R*_p_ values of N80 and 26Cr3Mo gradually increased, suggesting corrosion rate reduction due to biocide treatment. After the 1-h treatment, 200 ppm Biotreat 5475, 200 ppm Biotreat 5475 + 200 nM Peptide A, and 400 ppm Biotreat 5475 achieved 24%, 31%, and 43% 1/*R*_p_ reductions, respectively for N80. For 26Cr3Mo, the reductions were 34%, 41%, and 40%, respectively. The cocktail of 200 ppm Biotreat 5475 + 200 nM Peptide A led to an extra 1/*R*_p_ reduction for both N80 and 26Cr3Mo compared to 200 ppm Biotreat 5475 alone.

**Figure 7:**
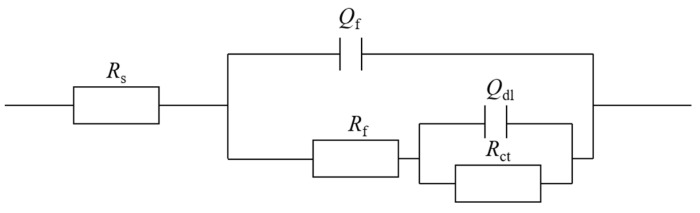
Equivalent circuit is employed to model EIS spectra.

**Figure 8:**
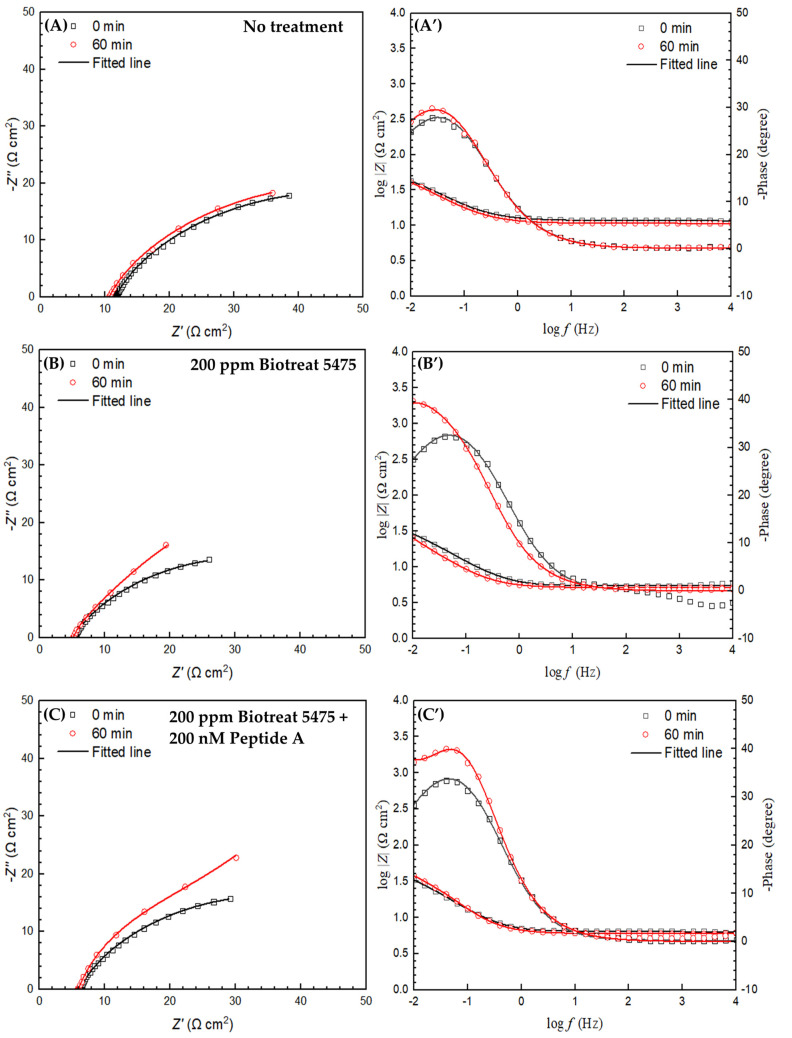

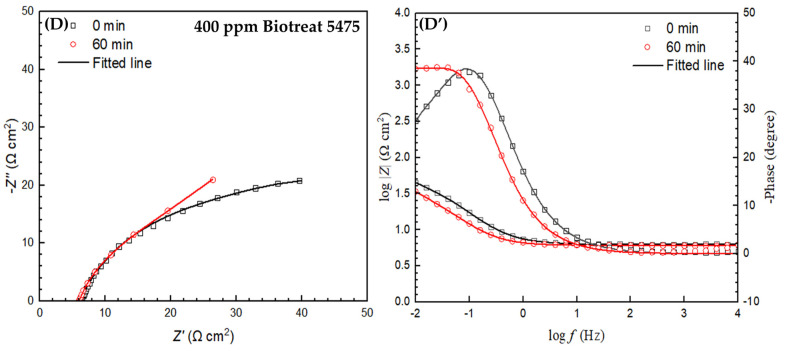
N80 Nyquist (**left**) and Bode (**right**) plots after 1-h biocide treatment: (**A**,**A’**) no treatment, (**B**,**B’**) 200 ppm Biotreat 5475, (**C**,**C’**) 200 ppm Biotreat 5475 + 200 nM Peptide A, and (**D**,**D’**) 400 ppm Biotreat 5475. (Phase curves start from 0 degree).

**Figure 9:**
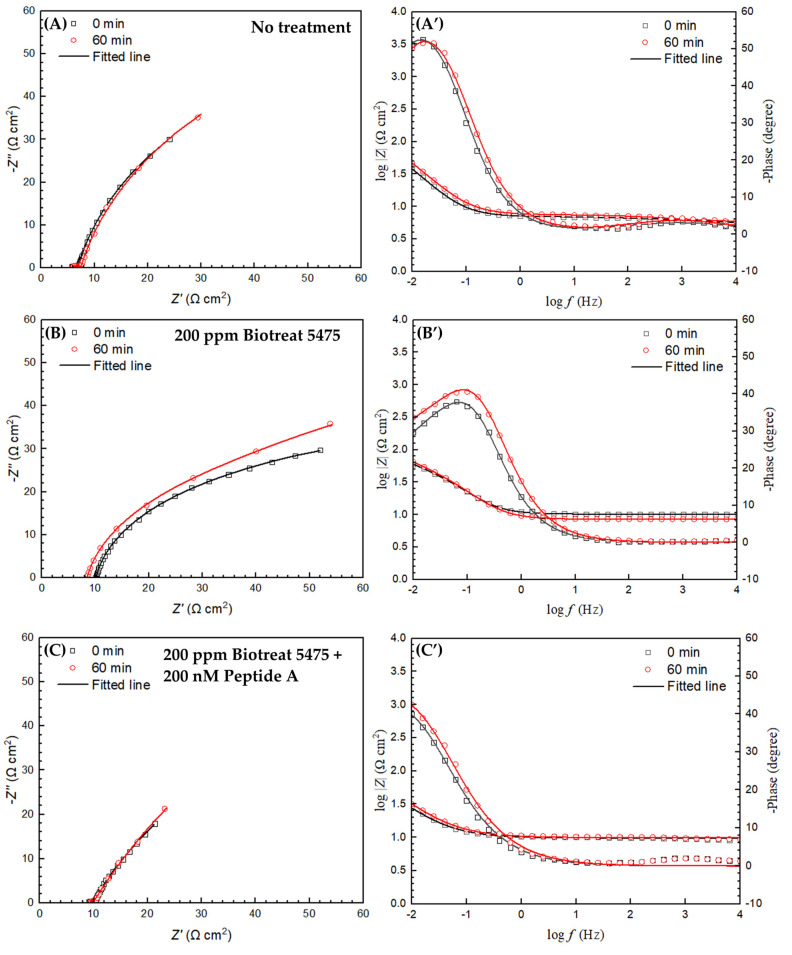

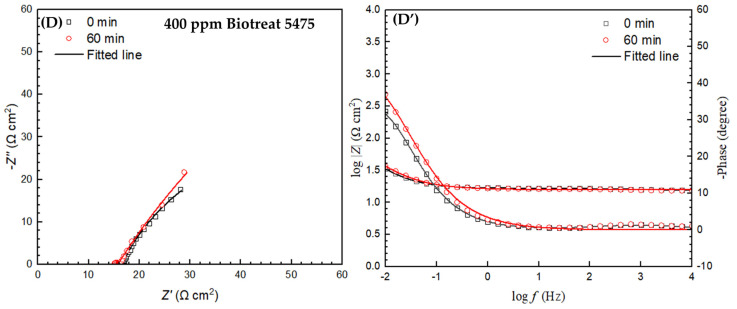
26Cr3Mo Nyquist (**left**) and Bode (**right**) plots after 1-h biocide treatment: (**A**,**A’**) no treatment, (**B**,**B’**) 200 ppm Biotreat 5475, (**C**,**C’**) 200 ppm Biotreat 5475 + 200 nM Peptide A, and (**D**,**D’**) 400 ppm Biotreat 5475. (Phase curves start from 0 degree).

The equivalent electrical circuit model shown in Figure 7 was used for EIS spectra modeling. *R*_s_ and *R*_f_ are solution resistance and resistance of the film consisting of the biofilm and the corrosion products, respectively. *R*_ct_ denotes charge transfer resistance. *Q*_f_ and *Q*_dl_ stand for the capacitance of the biofilm/corrosion product film and the double-layer capacitance, respectively. Nyquist and Bode plots of N80 and 26Cr3Mo are displayed in Figure 8 and Figure 9. The Nyquist plots in Figure 8A–D and Figure 9A–D indicate that biocide treatment increased the semi-circle diameters, indicating increased corrosion resistance. The EIS fitting results are listed in Table 1 and Table 2.

(*R*_ct_ + *R*_f_) is often used to estimate corrosion resistance that is comparable to *R*_p_ [56,57]. Because its inverse can represent corrosion rate, 1/(*R*_ct_ + *R*_f_) decrease is used to estimate corrosion inhibition efficacy for a biocide treatment,

(4) $$\eta_{i,\mathrm{EIS}}=\left(1-\frac{R_{\mathrm{ct},0}+R_{f,0}}{R_{\mathrm{ct}}+R_{f}}\right)\times 100\%$$

**Figure 10:**
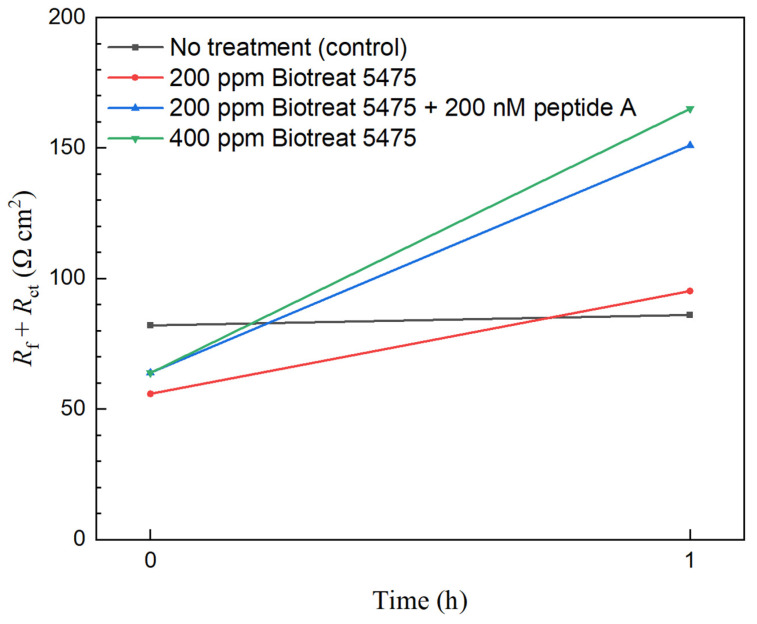
N80 *R*_f_ + *R*_ct_ curves from EIS modeling during 1-h time after biocide injection.

**Figure 11:**
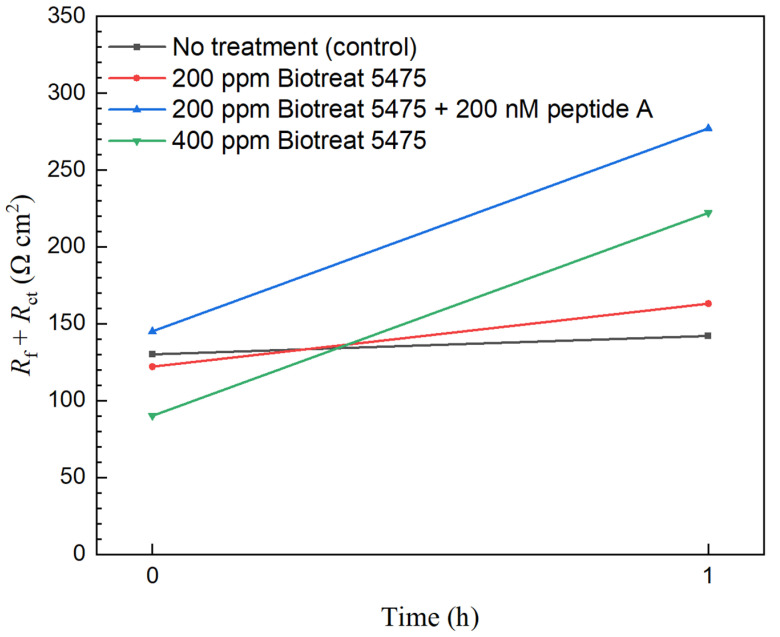
26Cr3Mo *R*_f_ + *R*_ct_ curves from EIS modeling during 1-h time after biocide injection.

In Figure 10 and Figure 11, (*R*_ct_ + *R*_f_) was stable for N80 and 26Cr3Mo during the 1-h period without biocide injection. One h after the biocide injection, (*R*_ct_ + *R*_f_) of both N80 and 26Cr3Mo increased significantly, suggesting decreased corrosion rates due to biocide injection. Based on fitted EIS parameters in Table 1 and Table 2, for N80, the 1-h biocide treatment with 200 ppm Biotreat 5475, 200 ppm Biotreat 5475 + 200 nM Peptide A, and 400 ppm Biotreat 5475 reduced 1/(*R*_ct_ + *R*_f_) by 41%, 58%, and 61%, respectively. For 26Cr3Mo, the reductions were 25%, 28%, and 60%, respectively. The EIS results indicated that the efficiency sequence was 200 ppm Biotreat 5475 < 200 ppm Biotreat 5475 + 200 nM Peptide A < 400 ppm Biotreat 5475, which is consistent with LPR results.

**Figure 12:**
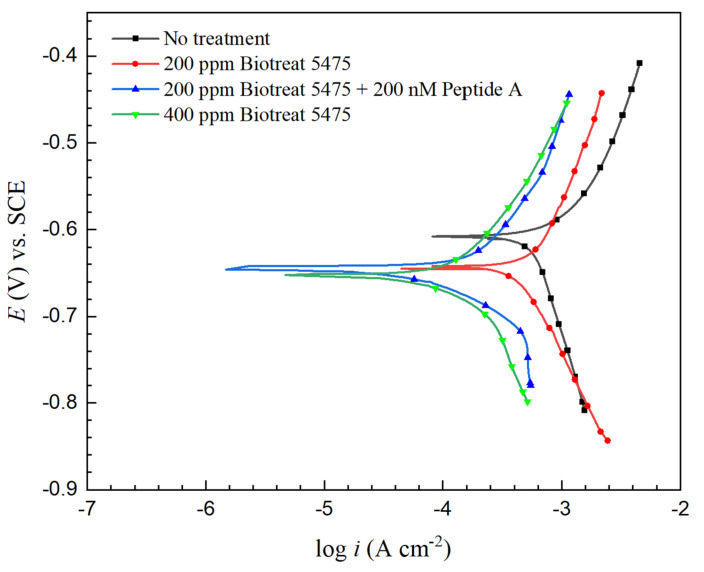
N80 potentiodynamic polarization curves of obtained 1.5 h after biocide injection into 3-d SRB broths.

**Figure 13:**
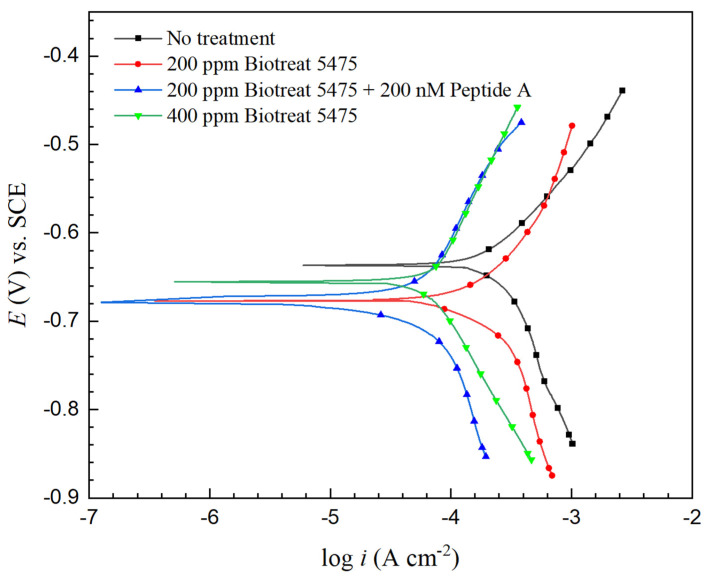
26Cr3Mo potentiodynamic polarization curves of obtained 1.5 h after biocide injection into 3-d SRB broths.

Figure 12 and Figure 13 present Tafel curves of N80 and 26Cr3Mo after biocide treatment with Tafel scans starting 1.5 h after the biocide injection. The fitted Tafel parameters are summarized in Table 3 and Table 4. A higher corrosion current density (*i*_corr_) correlates to a higher uniform corrosion rate. Without biocide injection, the *i*_corr_ values were 0.54 mA/cm^2^ and 0.26 mA/cm^2^ for N80 and 26Cr3Mo. A lower *i*_corr_ value of 26Cr3Mo also suggests that 26Cr3Mo had a greater (uniform) corrosion resistance than N80.

Corrosion inhibition efficiency (*η*_i_) calculated from *i*_corr_ is shown in Table 5. After the 1-h biocide treatment, *η*_i_ values for N80 were 15%, 70%, and 72% for 200 ppm Biotreat 5475, 200 ppm Biotreat 5475 + 200 nM Peptide A, and 400 ppm Biotreat 5475, respectively. For 26Cr3Mo, the values were 27%, 79%, and 75%, respectively. The 200 ppm Biotreat 5475 didn’t achieve a high corrosion inhibition, but 200 nM peptide turned out to enhance the biocide efficacy considerably, reaching a similar corrosion inhibition outcome as 400 ppm Biotreat 5475 did, which means cutting THPS dosage by half, consistent with the trend in sessile cell counts.

Table 5 summarizes the biocide efficacy data from sessile cell reduction, LPR 1/*R*_p_ reduction, EIS 1/(*R*_ct_ + *R*_f_) reduction, and PDP *i*_corr_ reduction. The data in this work indicated that all three electrochemical methods correctly supported sessile cell reduction outcomes. Table 5 data indicate that biocide treatment efficacies were similar for the two metals. Although different electrochemical methods provided different corrosion inhibition efficacy values, their trends were consistent. Thus, LPR, EIS, and PDP were all able to support the sessile cell count trend.

The findings in this work were comparable to other Peptide A studies in that Peptide A turned out to be an effective biocide enhancer that was able to cut the biocide dosage by nearly half [47,48,52]. Peptide A was found attractive as it functioned at a very low concentration (sub-ppm level). The biofilm dispersal effect appeared crucial in preventing biofilm formation and MIC mitigation. The mechanism of the biofilm dispersal effect of Peptide A was speculated in a previous study. It will be worthwhile to investigate it in depth in the future.

## 3. Materials and Methods

### 3.1. Bacterium, Metal, and Chemicals

Table 6 shows the elemental compositions of 26Cr3Mo and N80 steels. Biotreat 5475 was an industrial biocide from Clariant (Muttenz, Switzerland) that contained 75–80% of THPS by mass. Peptide A with 97.2% purity (based on peak area analysis in reverse-phase high-performance liquid chromatography) according to the supplier (Bachem Holding AG, Bubendorf, Switzerland) was used in this work. Table 7 and Table 8 display the test matrices for this work. *D. ferrophilus* (strain IS5), a highly-corrosive pure strain SRB [49,58], was immersed in enriched artificial seawater (EASW) culture medium inoculated with *D. ferrophilus* at 28 °C for 3 days to yield mature biofilms on the metal coupon surfaces. The composition of EASW is listed in Table 9. The pre-cut 26Cr3Mo and N80 coupons were coated with an inert liquid Epoxy coating (3M Product 323) except for a 10 mm × 10 mm exposed top work surface. The coupons were left at room temperature for drying overnight. The top surface was polished to 600 grit and the coupon was sterilized with anhydrous isopropanol before testing. The initial pH of EASW was adjusted to 7.0 using 5% (w/w) NaOH. The medium was sterilized in an autoclave at 121 °C for 20 min. Then, it was sparged with filter-sterilized N_2_ for 1 h to get rid of dissolved oxygen. Finally, an oxygen scavenger L-cysteine was added into the medium to reach a final concentration of 100 ppm. The biocide stock solution was filter-sterilized using a 0.22 μm Stericup single-use sterile filter (Millipore, Bedford, MA, USA). The inoculation (with a 1:100 volume ratio for inoculum vs. culture medium) was carried out in an anaerobic chamber filled with N_2_.

### 3.2. Sessile Cell Counts

For counting sessile cells, coupons of each metal type were put into separate 125 mL anaerobic vials with 50 mL EASW. After the 3-d pre-growth of *D. ferrophilus*, all coupons were taken out in the anaerobic chamber and rinsed with pH 7.4 phosphate-buffered saline (PBS) to remove planktonic cells. The coupons were then transferred into Petri dishes containing the pH 7.4 PBS solutions with and without biocide chemicals (1 cm^2^ coupon surface per 25 mL PBS) in the anaerobic chamber. After the 1-h biocide soaking treatment, sessile cells, which were motile, were counted using a hemocytometer under a 400× microscope [25].

### 3.3. Electrochemical Measurements

**Figure 14:**
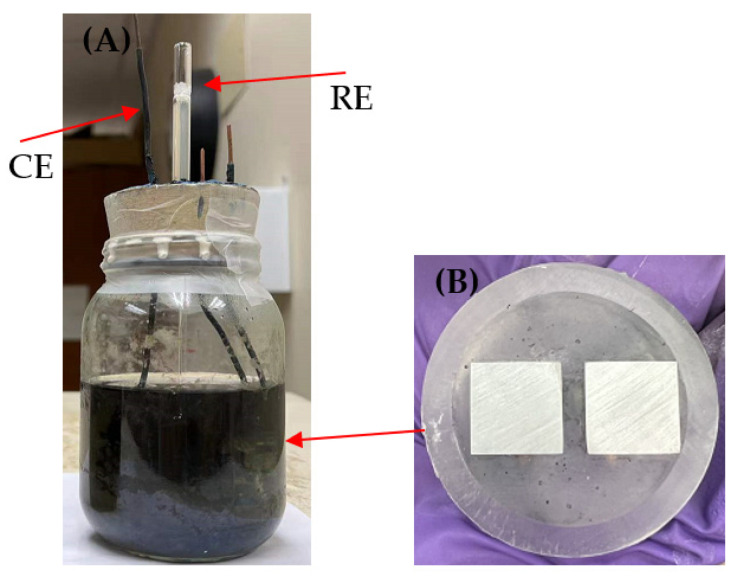
(**A**) Electrochemical cell right after SRB inoculation, and (**B**) dual coupons (each with a 1 cm^2^ surface area) in one working electrode Epoxy cake to provide replicate scans.

Electrochemical tests were performed in 450 mL glass cells, each filled with 250 mL EASW. For each metal type (N80 or 26Cr3Mo), 4 glass cells were used (1 for no-biocide control, 3 for 3 different biocide treatments). A three-electrode setup was adopted for measurements (Figure 14A). The working electrode (WE) Epoxy cake contained two replicate coupons as replicates (Figure 14B). The counter electrode (CE) was a thin platinum sheet, and a saturated calomel electrode (SCE) was used as the reference electrode (RE). Electrochemical measurements were carried out using a PCI4/750 potentiostat (Gamry Instruments, Inc., Warminster, PA, USA). OCP, LPR, EIS and PDP were measured in this work. LPR was measured at a scanning rate of 0.167 mV/s from −10 mV to 10 mV (vs. OCP) after OCP became stable. EIS was scanned from 10^5^ Hz to 0.01 Hz with a 10 mV amplitude sinusoidal signal. PDP was measured at the end of incubation. Tafel curves were obtained from two half-scans on the same working electrode starting from 0 mV to −200 mV (vs. OCP) and 0 mV to +200 mV (vs. OCP) with a scanning rate of 0.167 mV/s.

In the electrochemical tests, *D. ferrophilus* biofilms were pre-grown on the working electrode surfaces to reach maturity, which took 3 days of incubation. After that, a concentrated biocide stock solution was injected into the anaerobic glass cell. The glass cell was then gently shaken for 3 min to disperse the biocide. OCP and LPR were measured every 20 min during the 1 h following the biocide injection. EIS scan was conducted just before and after the 1-h biocide treatment to evaluate the biofilm’s response to the biocide treatment. Tafel scans were performed after other electrochemical measurements were performed. To evaluate corrosion inhibition efficiency (*η*_i_), reduction in *i*_corr_ was calculated using the following equation [52]:

(5) $$\eta_{i}=\left(1-\frac{i_{\mathrm{corr}}}{i_{\mathrm{corr},0}}\right)\times 100\%$$

where *i*_corr_ and *i*_corr,0_ represent corrosion current densities with and without biocide treatment, respectively. *i*_corr,0_ was obtained in the biotic control glass cell.

The biofilm kill test by soaking coupons in a biocide solution simulates a concentrated biocide plug between two pigs moving down a pipeline. Please note that corrosion weight loss change and corrosion pit depth change in this kind of 1-h (to simulate 1 h contact time or residence time) biofilm kill test are not measurable. Electrochemical tests are the only methods that can provide near-real time corrosion rate changes for biocide efficacy assessment to support sessile cell count reduction data.

## 4. Conclusions

26Cr3Mo showed a higher uniform corrosion resistance compared to N80 under the same conditions based on *R*_p_ and *i*_corr_ values before biocide treatment. The SRB biofilms on both metals responded similarly to Biotreat 5475 treatment. The various electrochemical corrosion measurements indicated that 200 ppm Biotreat 5475 was able to mitigate MIC by *D. ferrophilus*, but it was not very effective. The addition of 200 nM Peptide A considerably enhanced the efficacy of 200 ppm Biotreat 5475, which achieved similar efficacy as 400 ppm Biotreat 5475. This indicates that the addition of 200 nM Peptide cut the THPS dosage by nearly half. This work demonstrated that LPR, EIS, and PDP are reliable in assessing antimicrobial efficacy. They provided corrosion rate information that supported sessile cell reductions after biocide treatment in the 1-h biofilm kill test in which weight loss and pit depth changes were not measurable.

## Figures and Tables

**Table 1:** Fitted electrochemical parameters of N80 from EIS data in Figure 8.

	*t* (h)	*R*_s_ (Ω cm^2^)	*Q*_f_ (Ω^−1^ s^n^ cm^−2^)		*n*_1_	*R*_f_ (Ω cm^2^)	*Q*_dl_ (Ω^−1^ s^n^ cm^−2^)	*n*_2_	*R*_ct_ (Ω cm^2^)	*R*_f_ + *R*_ct_ (Ω cm^2^)
No treatment	0	11.5	4.1 × 10^−2^	0.41		0.28	8.6 × 10^−2^	0.79	82.0	82.0
	1	10.5	4.9 × 10^−2^	0.44		0.25	9.3 × 10^−2^	0.80	86.0	86.0
200 ppm Biotreat 5475	0	5.52	7.2 × 10^−2^	0.98		1.69	0.12	0.55	54.1	55.8
	1	5.16	0.22	0.79		15.2	0.18	0.64	80.0	95.2
200 ppm Biotreat 5475 + 200 nM Peptide A	0	6.32	0.10	0.55		0.17	6.0 × 10^−2^	0.98	63.7	63.9
	1	5.98	0.14	0.80		42.3	0.43	0.83	109	151
400 ppm Biotreat 5475	0	6.24	9.6 × 10^−2^	0.79		44.9	0.87	0.98	18.9	63.8
	1	6.01	0.16	0.81		31.4	0.27	0.70	134	165

**Table 2:** Fitted electrochemical parameters of 26Cr3Mo from EIS data in Figure 9.

	*t* (h)	*R*_s_ (Ω cm^2^)	*Q*_f_ (Ω^−1^ s^n^ cm^−2^)	*n*_1_	*R*_f_ (Ω cm^2^)	*Q*_dl_ (Ω^−1^ s^n^ cm^−2^)	*n*_2_	*R*_ct_ (Ω cm^2^)	*R*_f_ + *R*_ct_ (Ω cm^2^)
No treatment	0	5.38	9.9 × 10^−3^	0.49	1.66	0.27	0.87	128	130
	1	5.59	6.5 × 10^−3^	0.53	1.93	0.20	0.85	140	142
200 ppm Biotreat 5475	0	10.2	8.0 × 10^−2^	0.89	36.9	0.13	0.61	84.9	122
	1	8.46	7.1 × 10^−2^	0.87	44.1	0.12	0.65	119	163
200 ppm Biotreat 5475 + 200 nM Peptide A	0	15.3	1.3 × 10^−6^	0.39	12.1	0.32	0.72	133	145
	1	15.8	0.25	0.69	0.01	8.2 × 10^−13^	0.79	277	277
400 ppm Biotreat 5475	0	9.73	0.35	0.77	88.6	3.9 × 10^−3^	0.61	1.37	90
	1	9.87	0.26	0.70	222	0.16	0.76	0.01	222

**Table 3:** Tafel parameters of N80 fitted from potentiodynamic polarization curves in Figure 12.

	*β*_a_ (V/dec)	*β*_c_ (V/dec)	*E*_corr_ (V) vs. SCE	*i*_corr_ (mA/cm^2^)
No treatment	0.103	−0.440	−0.611	0.54
200 ppm Biotreat 5475	0.205	−0.287	−0.645	0.46
200 ppm Biotreat 5475 + 200 nM Peptide A	0.161	−0.148	−0.649	0.16
400 ppm Biotreat 5475	0.200	−0.192	−0.656	0.15

**Table 4:** Tafel parameters of 26Cr3Mo fitted from potentiodynamic polarization curves in Figure 13.

	*β*_a_ (V/dec)	*β*_c_ (V/dec)	*E*_corr_ (V) vs. SCE	*i*_corr_ (mA/cm^2^)
No treatment	0.191	−0.271	−0.647	0.26
200 ppm Biotreat 5475	0.208	−0.301	−0.675	0.19
200 ppm Biotreat 5475 + 200 nM Peptide A	0.286	−0.242	−0.677	0.055
400 ppm Biotreat 5475	0.287	−0.245	−0.659	0.066

**Table 5:** Biocide efficacy data from sessile cell reduction, and estimated MIC inhibition efficiencies from various electrochemical tests.

			Corrosion Inhibition Efficiency					
	Sessile Cell Count Reduction		LPR 1/*R*_p_ Reduction (*η*_i,LPR_)		EIS 1/(*R*_ct_ + *R*_f_) Reduction (*η*_i,EIS_)		PDP *i*_corr_ Reduction (*η*_i_)	
	N80	26Cr3Mo	N80	26Cr3Mo	N80	26Cr3Mo	N80	26Cr3Mo
200 ppm Biotreat 5475	68.2%	78.0%	24%	34%	41%	25%	15%	27%
200 ppm Biotreat 5475 + 200 nM Peptide A	97.8%	98.0%	31%	41%	58%	48%	70%	79%
400 ppm Biotreat 5475	98.7%	98.3%	43%	40%	61%	60%	72%	75%

**Table 6:** Elemental compositions (wt. %) of N80 [59] and 26Cr3Mo (Fe balance).

Metal	C	Mn	Cr	Ni	Cu	P	S	Si	Mo	V
N80	0.34–0.38	1.45–1.70	0.15			0.020	0.015	0.20–0.35		
26Cr3Mo	0.25	0.49	2.99	0.02	0.02	0.008	0.001	0.26	0.12	0.015

**Table 7:** Test matrix for 1-h SRB biofilm kill test in Petri dishes after 3-d pre-growth.

Parameter	Condition
Microbe	*D. ferrophilus* IS5
Cultural medium	EASW
Coupon material	N80 and 26Cr3Mo
Liquid volume	50 mL in 125 mL anaerobic vials
Inoculum size	0.5 mL in anaerobic vials
Treatment method	No treatment control 200 ppm Biotreat 5475 200 ppm Biotreat 5475 + 200 nM Peptide A 400 ppm Biotreat 5475 1 cm^2^ coupon surface/25 mL liquid
Temperature	28 °C
Initial pH	7.0 ± 0.2
Incubation time before biocide soaking	3 d
Biocide treatment time	1 h in Petri dish in an anaerobic chamber
Assay	Sessile cell counts on coupons

**Table 8:** Test matrix for electrochemical responses to biocide injection into 3-d SRB broth.

Parameter	Condition
Microbe	*D. ferrophilus* IS5
Cultural medium	EASW
Coupon material	N80 and 26Cr3Mo
Liquid volume	250 mL in 450 mL glass cells
Inoculum size	2.5 mL in glass cells
Treatment method	No treatment control 200 ppm Biotreat 5475 200 ppm Biotreat 5475 + 200 nM Peptide A 400 ppm Biotreat 5475 4 glass cells used. Injection was followed by 3 min gentle shaking to dispere biocide.
Temperature	28 °C
Initial pH	7.0 ± 0.2
Incubation time before biocide injection	3 d
Biocide treatment time	1 h
Assay	OCP, LPR, EIS, and Tafel scans using glass cells

**Table 9:** Composition of EASW culture medium [52].

Chemical	Amount
NaCl	23.476 g
Na_2_SO_4_	3.917 g
NaHCO_3_	0.192 g
KCl	0.664 g
KBr	0.096 g
H_3_BO_3_	0.026 g
MgCl_2_	4.965 g
SrCl_2_·6H_2_O	0.04 g
CaCl_2_·2H_2_O	1.469 g
Sodium lactate	3.5 g
Yeast extract	1.0 g
Sodium citrate	0.5 g
MgSO_4_·7H_2_O	0.71 g
CaSO_4_·2H_2_O	0.1 g
NH_4_Cl	0.1 g
K_2_HPO_4_	0.05 g
Fe(NH_4_)_2_(SO_4_)_2_·6H_2_O	1.37 g
DI water	1 L

## Data Availability

The data presented in this work are available on request from the corresponding author.

## References

[1] Xu D., Gu T., Lovley D.R. (2023). Microbially Mediated Metal Corrosion. Nat. Rev. Microbiol..

[2] Zhu S.D., Fu A.Q., Miao J., Yin Z.F., Zhou G.S., Wei J.F. (2011). Corrosion of N80 Carbon Steel in Oil Field Formation Water Containing CO2 in the Absence and Presence of Acetic Acid. Corros. Sci..

[3] Ura-Bińczyk E., Banaś J., Mazurkiewicz B., Solarski W., Lewandowska M., Roguska A., Andrzejczuk M., Balcer M., Kulik S., Żarnowiec P. (2019). On-Site Monitoring and Laboratory Characterization of Corrosion Processes in the Geothermal Water of Polish Lowland. Geothermics.

[4] Senthilmurugan B., Radhakrishnan J.S., Poulsen M., Tang L., AlSaber S. (2021). Assessment of Microbiologically Influenced Corrosion in Oilfield Water Handling Systems Using Molecular Microbiology Methods. Upstream Oil Gas Technol..

[5] Sun J., Sun C., Wang Y. (2016). Effect of Cr Content on the Electrochemical Behavior of Low- Chromium X65 Steel in CO_2_ Environment. Int. J. Electrochem. Sci..

[6] Xu L., Wang B., Zhu J., Li W., Zheng Z. (2016). Effect of Cr Content on the Corrosion Performance of Low-Cr Alloy Steel in a CO_2_ Environment. Appl. Surf. Sci..

[7] Hua Y., Mohammed S., Barker R., Neville A. (2020). Comparisons of Corrosion Behaviour for X65 and Low Cr Steels in High Pressure CO2-Saturated Brine. J. Mater. Sci. Technol..

[8] Lin X., Liu W., Wu F., Xu C., Dou J., Lu M. (2015). Effect of O_2_ on Corrosion of 3Cr Steel in High Temperature and High Pressure CO_2_–O_2_ Environment. Appl. Surf. Sci..

[9] Guo S., Xu L., Zhang L., Chang W., Lu M. (2012). Corrosion of Alloy Steels Containing 2% Chromium in CO_2_ Environments. Corros. Sci..

[10] Kamimura T., Stratmann M. (2001). The Influence of Chromium on the Atmospheric Corrosion of Steel. Corros. Sci..

[11] Ma Y., Zhang Y., Zhang R., Guan F., Hou B., Duan J. (2020). Microbiologically Influenced Corrosion of Marine Steels within the Interaction between Steel and Biofilms: A Brief View. Appl. Microbiol. Biotechnol..

[12] L80-3Cr Tubing Pup Joint EUE PIN X BOX. http://www.forwellequip.com/products/L80-3Cr-Tubing-Pup-Joint-EUE-PIN-X-BOX.html.

[13] Gaines R.H. (1910). Bacterial Activity as a Corrosive Influence in the Soil. J. Ind. Eng. Chem..

[14] Williamson C.H.D., Jain L.A., Mishra B., Olson D.L., Spear J.R. (2015). Microbially Influenced Corrosion Communities Associated with Fuel-Grade Ethanol Environments. Appl. Microbiol. Biotechnol..

[15] Bolton N., Critchley M., Fabien R., Cromar N., Fallowfield H. (2010). Microbially Influenced Corrosion of Galvanized Steel Pipes in Aerobic Water Systems. J. Appl. Microbiol..

[16] Dong Y., Lekbach Y., Li Z., Xu D., El Abed S., Ibnsouda Koraichi S., Wang F. (2020). Microbiologically Influenced Corrosion of 304L Stainless Steel Caused by an Alga Associated Bacterium *Halomonas titanicae*. J. Mater. Sci. Technol..

[17] Koch G.H., Brongers M.P., Thompson N.G., Virmani Y.P., Payer J.H. (2002). Corrosion Cost and Preventive Strategies in the United States.

[18] Fu Q., Wei B., Xu J., Qin Q., Bai Y., Yu C., Sun C. (2023). Corrosion Mechanism of Pseudomonas Stutzeri on X80 Steel Subjected to Desulfovibrio Desulfuricans under Elastic Stress and Yield Stress. Corros. Sci..

[19] Videla H.A., Herrera L.K. (2005). Microbiologically Influenced Corrosion: Looking to the Future. Int. Microbiol..

[20] Abedi S.S., Abdolmaleki A., Adibi N. (2007). Failure Analysis of SCC and SRB Induced Cracking of a Transmission Oil Products Pipeline. Eng. Fail. Anal..

[21] Cai Z., Xu J., Wei B., Sun C. (2022). A Comparative Study of Sulfate-Reducing *Desulfovibrio desulfuricans* Induced Corrosion Behaviors in Q235, X65, X70, and X80 Pipeline Steels. Int. J. Press. Vessels Pip..

[22] Scarascia G., Wang T., Hong P.-Y. (2016). Quorum Sensing and the Use of Quorum Quenchers as Natural Biocides to Inhibit Sulfate-Reducing Bacteria. Antibiotics.

[23] Little B., Lee J., Ray R. (2007). A Review of ‘Green’ Strategies to Prevent or Mitigate Microbiologically Influenced Corrosion. Biofouling.

[24] Md Zain W.S., Hairul Salleh N.I., Abdullah A. (2018). Natural Biocides for Mitigation of Sulphate Reducing Bacteria. Int. J. Corros..

[25] Wang D., Kijkla P., Mohamed M.E., Saleh M.A., Kumseranee S., Punpruk S., Gu T. (2021). Aggressive Corrosion of Carbon Steel by *Desulfovibrio ferrophilus* IS5 Biofilm Was Further Accelerated by Riboflavin. Bioelectrochemistry.

[26] Mah T.-F.C., O’Toole G.A. (2001). Mechanisms of Biofilm Resistance to Antimicrobial Agents. Trends Microbiol..

[27] Tripathi A.K., Thakur P., Saxena P., Rauniyar S., Gopalakrishnan V., Singh R.N., Gadhamshetty V., Gnimpieba E.Z., Jasthi B.K., Sani R.K. (2021). Gene Sets and Mechanisms of Sulfate-Reducing Bacteria Biofilm Formation and Quorum Sensing With Impact on Corrosion. Front. Microbiol..

[28] Akshaya S., Rowlo P.K., Dukle A., Nathanael A.J. (2022). Antibacterial Coatings for Titanium Implants: Recent Trends and Future Perspectives. Antibiotics.

[29] Viera M.R., Guiamet P.S., De Mele M.F.L., Videla H.A. (1999). Biocidal Action of Ozone against Planktonic and Sessile *Pseudomonas fluorescens*. Biofouling.

[30] Grande Burgos M.J., Lucas López R., López Aguayo M.D.C., Pérez Pulido R., Gálvez A. (2013). Inhibition of Planktonic and Sessile Salmonella Enterica Cells by Combinations of Enterocin AS-48, Polymyxin B and Biocides. Food Control.

[31] Iñiguez-Moreno M., Gutiérrez-Lomelí M., Avila-Novoa M.G. (2021). Removal of Mixed-Species Biofilms Developed on Food Contact Surfaces with a Mixture of Enzymes and Chemical Agents. Antibiotics.

[32] Flemming H.-C., Heitz E., Sand W., Flemming H.C. (1996). Biofouling and Microbiologically Influenced Corrosion (MIC)-an Economical and Technical Overview. Microbial Deterioration of Materials.

[33] Zuo R. (2007). Biofilms: Strategies for Metal Corrosion Inhibition Employing Microorganisms. Appl. Microbiol. Biotechnol..

[34] Kampf G. (2019). Antibiotic Resistance Can Be Enhanced in Gram-Positive Species by Some Biocidal Agents Used for Disinfection. Antibiotics.

[35] Kampf G. (2018). Biocidal Agents Used for Disinfection Can Enhance Antibiotic Resistance in Gram-Negative Species. Antibiotics.

[36] Wang D., Unsal T., Kumseranee S., Punpruk S., Saleh M.A., Alotaibi M.D., Xu D., Gu T. (2022). Mitigation of Carbon Steel Biocorrosion Using a Green Biocide Enhanced by a Nature-Mimicking Anti-Biofilm Peptide in a Flow Loop. Bioresour. Bioprocess..

[37] Di Martino P. (2021). Ways to Improve Biocides for Metalworking Fluid. AIMS Microbiol..

[38] Sharma M., Liu H., Chen S., Cheng F., Voordouw G., Gieg L. (2018). Effect of Selected Biocides on Microbiologically Influenced Corrosion Caused by *Desulfovibrio ferrophilus* IS5. Sci. Rep..

[39] Conlette O. (2014). Impacts of Tetrakis-Hydroxymethyl Phosphonium Sulfate (THPS) Based Biocides on the Functional Group Activities of Some Oil Field Microorganisms Associated with Corrosion and Souring. Br. Microbiol. Res. J..

[40] Silva P., Oliveira S.H., Vinhas G.M., Carvalho L.J., Barauna O.S., Urtiga Filho S.L., Lima M.A.G. (2021). Tetrakis Hydroxymethyl Phosphonium Sulfate (THPS) with Biopolymer as Strategy for the Control of Microbiologically Influenced Corrosion in a Dynamic System. Chem. Eng. Process. Process Intensif..

[41] Sharma M., Menon P., Voordouw J., Shen Y., Voordouw G. (2018). Effect of Long Term Application of Tetrakis (Hydroxymethyl) Phosphonium Sulfate (THPS) in a Light Oil-Producing Oilfield. Biofouling.

[42] Rüegg U.T., Rudinger J. (1977). Reductive Cleavage of Cystine Disulfides with Tributylphosphine. Methods in Enzymology.

[43] Parker A.J., Kharasch N. (1959). The Scission of the Sulfur-Sulfur Bond. Chem. Rev..

[44] Okoro C.C. (2015). The Biocidal Efficacy of Tetrakis-Hydroxymethyl Phosphonium Sulfate (THPS) Based Biocides on Oil Pipeline PigRuns Liquid Biofilms. Pet. Sci. Technol..

[45] Talbot R.E., Larsen J., Sanders P.F. Experience with the Use of Tetrakishydroxymethylphosphonium Sulfate (THPS) for the Control of Downhole Hydrogen Sulfide. Proceedings of the CORROSION/2000 Conference, Paper Number NACE-00123.

[46] Jia R., Li Y., Al-Mahamedh H.H., Gu T. (2017). Enhanced Biocide Treatments with D-Amino Acid Mixtures against a Biofilm Consortium from a Water Cooling Tower. Front. Microbiol..

[47] Wang D., Ramadan M., Kumseranee S., Punpruk S., Gu T. (2020). Mitigating Microbiologically Influenced Corrosion of an Oilfield Biofilm Consortium on Carbon Steel in Enriched Hydrotest Fluid Using 2,2-Dibromo-3-Nitrilopropionamide (DBNPA) Enhanced by a 14-Mer Peptide. J. Mater. Sci. Technol..

[48] Jia R., Yang D., Dou W., Liu J., Zlotkin A., Kumseranee S., Punpruk S., Li X., Gu T. (2019). A Sea Anemone-Inspired Small Synthetic Peptide at Sub-Ppm Concentrations Enhanced Biofilm Mitigation. Int. Biodeterior. Biodegrad..

[49] Xu L., Kijkla P., Kumseranee S., Punpruk S., Gu T. (2023). “Corrosion-Resistant” Chromium Steels for Oil and Gas Pipelines Can Suffer from Very Severe Pitting Corrosion by a Sulfate-Reducing Bacterium. J. Mater. Sci. Technol..

[50] Astuti D., Purwasena I.A., Putri F.Z. (2018). Potential of Biosurfactant as an Alternative Biocide to Control Biofilm Associated Biocorrosion. J. Environ. Sci. Technol..

[51] Bardouniotis E., Ceri H., Olson M.E. (2003). Biofilm Formation and Biocide Susceptibility Testing of Mycobacterium Fortuitum and Mycobacterium Marinum. Curr. Microbiol..

[52] Wang J., Liu H., Mohamed M.E.-S., Saleh M.A., Gu T. (2022). Mitigation of Sulfate Reducing *Desulfovibrio Ferrophilus* Microbiologically Influenced Corrosion of X80 Using THPS Biocide Enhanced by Peptide A. J. Mater. Sci. Technol..

[53] Ding Z., Tang Y., Liu L., Ding Z., Tan Y., He Q. (2022). Improving the Adhesive, Mechanical, Tribological Properties and Corrosion Resistance of Reactive Sputtered Tantalum Oxide Coating on Ti6Al4V Alloy via Introducing Multiple Interlayers. Ceram. Int..

[54] Lokesh K.S., De Keersmaecker M., Elia A., Depla D., Dubruel P., Vandenabeele P., Van Vlierberghe S., Adriaens A. (2012). Adsorption of Cobalt (II) 5,10,15,20-Tetrakis(2-Aminophenyl)-Porphyrin onto Copper Substrates: Characterization and Impedance Studies for Corrosion Inhibition. Corros. Sci..

[55] Tran T.T.T., Kannoorpatti K., Padovan A., Thennadil S. (2021). A Study of Bacteria Adhesion and Microbial Corrosion on Different Stainless Steels in Environment Containing *Desulfovibrio vulgaris*. R. Soc. Open Sci..

[56] Adama K., Onyeachu I. (2022). The Corrosion Characteristics of SS316L Stainless Steel in a Typical Acid Cleaning Solution and Its Inhibition by 1-Benzylimidazole: Weight Loss, Electrochemical and SEM Characterizations. J. Niger. Soc. Phys. Sci..

[57] Jin S., Amira S., Ghali E. (2007). Electrochemical Impedance Spectroscopy Evaluation of the Corrosion Behavior of Die Cast and Thixocast AXJ530 Magnesium Alloy in Chloride Solution. Adv. Eng. Mater..

[58] Dinh H.T., Kuever J., Mußmann M., Hassel A.W., Stratmann M., Widdel F. (2004). Iron Corrosion by Novel Anaerobic Microorganisms. Nature.

[59] *API 5CT N80*; (N80-1 and N80-Q Types) Casing Pipe—WLD Steel. API Steel Oilfield Pipeline Casing Pipe OCTG Manufacturer Supplier. https://www.wldsteel.com/product/api-5ct-n80-casing-pipe/.

